# Performance of externally validated machine learning models based on histopathology images for the diagnosis, classification, prognosis, or treatment outcome prediction in female breast cancer: A systematic review

**DOI:** 10.1016/j.jpi.2023.100348

**Published:** 2023-11-05

**Authors:** Ricardo Gonzalez, Peyman Nejat, Ashirbani Saha, Clinton J.V. Campbell, Andrew P. Norgan, Cynthia Lokker

**Affiliations:** a DeGroote School of Business, McMaster University, Hamilton, Ontario, Canada; b Division of Computational Pathology and Artificial Intelligence, Department of Laboratory Medicine and Pathology, Mayo Clinic, Rochester, MN, United States; c Department of Artificial Intelligence and Informatics, Mayo Clinic, Rochester, MN, United States; d Department of Oncology, Faculty of Health Sciences, McMaster University, Hamilton, Ontario, Canada; e Escarpment Cancer Research Institute, McMaster University and Hamilton Health Sciences, Hamilton, Ontario, Canada; f William Osler Health System, Brampton, Ontario, Canada; g Department of Pathology and Molecular Medicine, Faculty of Health Sciences, McMaster University, Hamilton, Ontario, Canada; h Department of Laboratory Medicine and Pathology, Mayo Clinic, Rochester, MN, United States; i Health Information Research Unit, Department of Health Research Methods, Evidence and Impact, McMaster University, Hamilton, Ontario, Canada

**Keywords:** Breast neoplasms, Pathology, Validation studies, Machine learning, Systematic review

## Abstract

Numerous machine learning (ML) models have been developed for breast cancer using various types of data. Successful external validation (EV) of ML models is important evidence of their generalizability. The aim of this systematic review was to assess the performance of externally validated ML models based on histopathology images for diagnosis, classification, prognosis, or treatment outcome prediction in female breast cancer. A systematic search of MEDLINE, EMBASE, CINAHL, IEEE, MICCAI, and SPIE conferences was performed for studies published between January 2010 and February 2022. The Prediction Model Risk of Bias Assessment Tool (PROBAST) was employed, and the results were narratively described. Of the 2011 non-duplicated citations, 8 journal articles and 2 conference proceedings met inclusion criteria. Three studies externally validated ML models for diagnosis, 4 for classification, 2 for prognosis, and 1 for both classification and prognosis. Most studies used Convolutional Neural Networks and one used logistic regression algorithms. For diagnostic/classification models, the most common performance metrics reported in the EV were accuracy and area under the curve, which were greater than 87% and 90%, respectively, using pathologists' annotations/diagnoses as ground truth. The hazard ratios in the EV of prognostic ML models were between 1.7 (95% CI, 1.2–2.6) and 1.8 (95% CI, 1.3–2.7) to predict distant disease-free survival; 1.91 (95% CI, 1.11–3.29) for recurrence, and between 0.09 (95% CI, 0.01–0.70) and 0.65 (95% CI, 0.43–0.98) for overall survival, using clinical data as ground truth. Despite EV being an important step before the clinical application of a ML model, it hasn't been performed routinely. The large variability in the training/validation datasets, methods, performance metrics, and reported information limited the comparison of the models and the analysis of their results. Increasing the availability of validation datasets and implementing standardized methods and reporting protocols may facilitate future analyses.

## Introduction

### Rationale

Female breast cancer is the most commonly diagnosed cancer and, in women, the most frequent cause of cancer mortality.^1^ Histopathological examination of breast tissue samples is the reference standard for cancer diagnosis and is used to determine the prognosis of a patient and risk factors to predict outcomes.^2^^,^^3^

Pathologists have been using microscopes with glass slides containing preserved human tissue to help make a diagnosis for more than 100 years. Now, these slides can be scanned and digitized to be viewed on computer screens.^4^^,^^5^ The process of scanning glass slides to produce digital images (Whole-Slide Images or WSI) was initially called “Digital Pathology” (DP).^4^ However, this term has evolved and is now used to encompass many other related processes in the modern pathology workflow.^4^^,^^5^ DP has been approved and implemented in laboratories for routine diagnosis in many countries and is expected to increase in the near future.^6^^,^^7^ Beyond other potential benefits, DP can facilitate the implementation of machine learning-based computational pathology tools to support diagnostic pathology workflows.^8^

ML model basic development steps include problem formulation,^9^ ML algorithm selection,^10^ data preparation,^10^^,^^11^ ML model training,^12^ hyperparameter tuning,^12^ model evaluation,^12^ and model deployment/maintenance.^9^^,^^10^ In a data-rich situation, the best approach to build a prediction/classification model is to randomly divide an input dataset into 3 parts: a training set, a validation set, and a testing set.^11^^,^^13^ The former is used to fit the model, the second to estimate the prediction error for model selection, and the later to assess the generalization error of the selected model.^13^ As the terminology employed to refer to ML models’ evaluation and validation datasets could confuse readers,^12^^,^^14^ the following terms are used here:

- Internal validation: Model evaluation conducted with data extracted from an input dataset (i.e., the “testing set” mentioned above)^11^^,^^15^, ^16^, ^17^ that was set aside from the training/tuning dataset at the beginning of the study to evaluate the final version of a ML model a single time.^11^
- EV: Model evaluation conducted with data extracted from independent datasets^11^^,^^15^, ^16^, ^17^ to evaluate the final version of a ML model a single time.^11^
- Training/Tuning datasets: Used for model training, optimization, and/or model selection.^11^

During tissue processing, slide preparation, slide digitization, image compression, and image storage, hidden variables (i.e., image data unrelated to the actual prediction/classification task that can affect the performance of ML models) may be introduced to WSIs.^18^, ^19^, ^20^ As protocols, equipment, and consumables utilized during these processes vary among different institutions,^21^ the independent datasets used during EV should ideally be extracted from a different data source, such as another clinic or hospital system.^11^^,^^15^, ^16^, ^17^

Only EV is considered important evidence of generalizability as patterns learned from hidden variables of training datasets (instead or in addition to those that can be learned from the intended target variables) are not expected to improve ML models’ performance when they are tested with independent datasets.^11^^,^^15^^,^^18^ Despite this, most of these models have not been externally validated.^16^^,^^17^^,^^22^, ^23^, ^24^, ^25^, ^26^, ^27^, ^28^

### Objective

This systematic review aimed to assess the performance of externally validated ML models based on histopathology images for diagnosis, classification, prognosis, or treatment outcome prediction in female breast cancer.

## Material and methods

### Eligibility criteria

We conducted a systematic review following the PRISMA 2020 guidelines.^29^ Studies focused on female breast cancer (invasive tumors or carcinomas in situ) that externally validated the performance of ML models using data extracted from histopathology images (stained with hematoxylin/eosin or other histochemical stains) directly for diagnosis, classification, prognosis, or treatment response prediction were included. The EV had to be suggested, implied, or mentioned in the Title/Abstract and conducted with breast cancer images for a study to be incorporated. In addition to those not meeting the above criteria, studies validating ML models developed to predict biomarkers were excluded.

### Information sources

A systematic search of original research journal papers and conference proceedings written in English and published from January 1, 2010 to February 28, 2022 was conducted in March 2022 using the following databases: MEDLINE (via OVID), EMBASE (via OVID), CINAHL (via EBSCO), IEEE (via IEEE Xplore), MICCAI (via Springer link), and SPIE conferences. A brief description of these databases is presented in Appendix A. The search strategies were developed by authors and reviewed by a health sciences librarian with expertise in systematic reviews.

### Search strategy

The search strategies are shown in Appendix B.

### Selection process

Search results were imported into Rayyan^30^ to remove duplicates and for title/abstract screening. Two reviewers independently conducted the initial title and abstract screening. Selected full-text articles were downloaded for all titles that met the inclusion criteria or where there was any uncertainty whether the inclusion criteria were met (e.g., when it was unclear if EV was performed). Two reviewers independently conducted the full-text screening using Mendeley.^31^ Additional information was requested from study authors when needed. A third person acted as an adjudicator to resolve any conflicts.

### Data collection process

One author independently extracted data from included studies with verification by a second.

### Data items

The following information was extracted: Authors, publication date, country of study, objectives, and main resus, including all performance measures in the EV of the ML models. In addition, related to the ML models, the following data were extracted: Algorithms employed; source, number, and type of images used for model development (training/tuning and internal validation datasets) and EV; histological type of tumors/entities contained in the training and EV datasets; details on preprocessing of the images; software platform(s) used for annotation/ground-truth preparation and computational purposes.

### Study risk of bias assessment

The risk of bias by using PROBAST (prediction model risk of bias assessment tool) for non-randomized studies was assessed by one author and confirmed by another.

### Effect measures

The effect measures were all performance metrics used to evaluate the ML model during EV, such as accuracy, area under the curve (AUC), precision, recall, etc.

### Synthesis methods

The main study characteristics and findings are presented in the text and summarized in tables. Results are narratively described. A meta-analysis was not conducted because of the heterogeneity of the studies in algorithms, type of images, and reported outcomes.

## Results

### Study selection

**Fig. 1:**
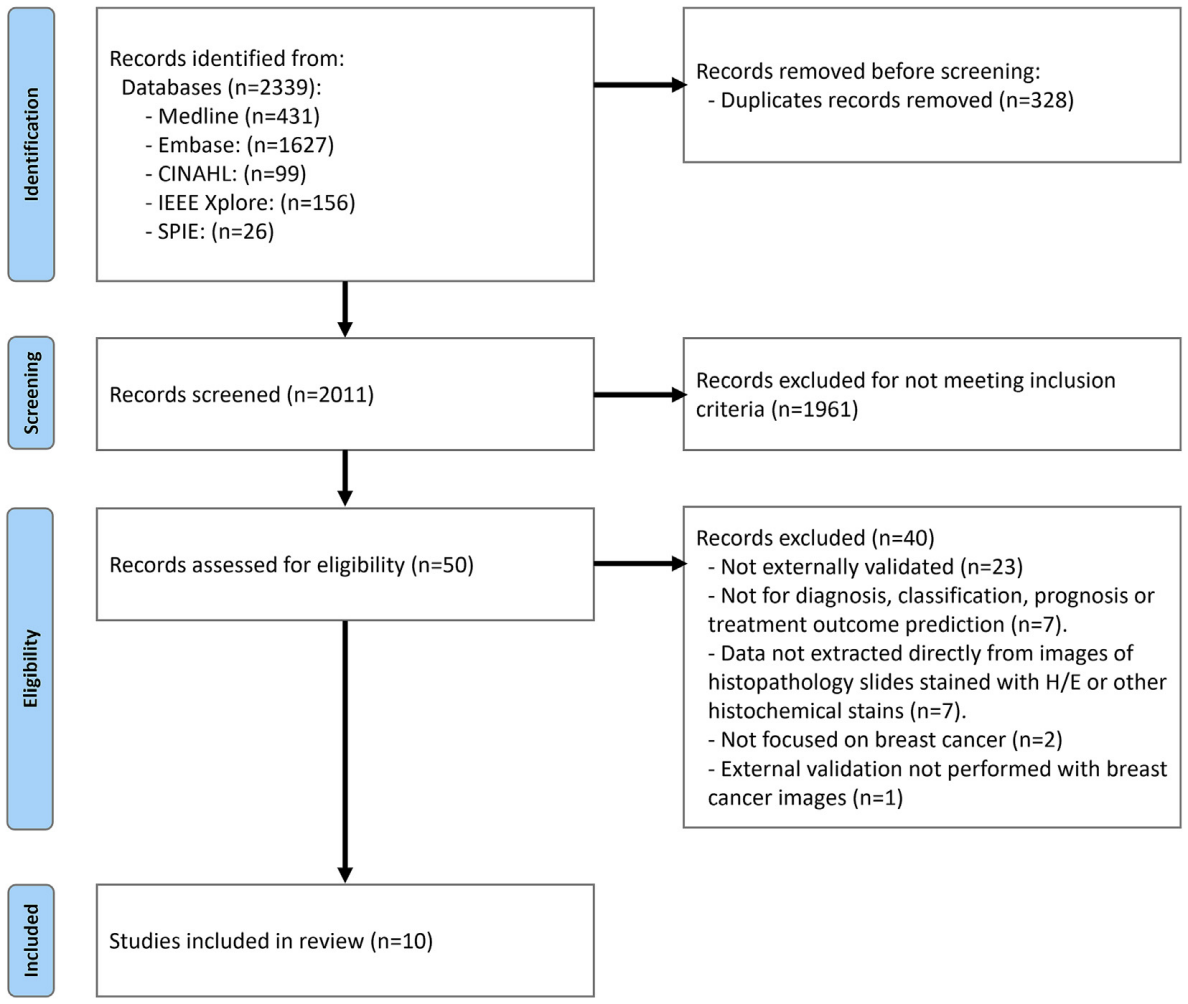
PRISMA flow diagram of the studies identification process for the systematic review.

The search queries identified 2157 articles and 182 conference proceedings. After removing 328 duplicates and excluding 1961 publications during the title and abstract screening, 50 were assessed during full-text screening, and 10 were included in the review (Fig. 1).

**Fig. 2:**
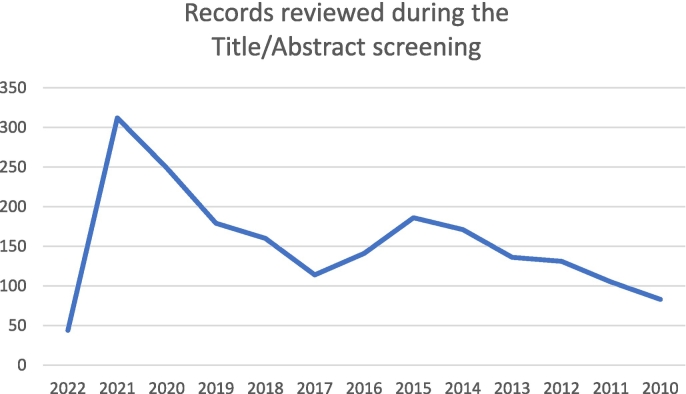
Distribution of the records reviewed during the Title/Abstract screening by year (January 1, 2010–February 28, 2022).

The number of records reviewed during the Title/Abstract screening progressively increased from 2010 to 2015, declined from 2015 to 2017, and rose again from 2017 to 2021. In 2022, only the records published from January 1 to February 28 are shown (Fig. 2).

Examples of records that passed and did not pass the Title/Abstract screening are provided in Appendix C.

### Study characteristics

In the included studies, 3 evaluated ML models for diagnostic purposes,^32^, ^33^, ^34^ 4 for classification purposes,^35^, ^36^, ^37^, ^38^ 2 for prognosis purposes,^39^^,^^40^ and 1 for prognostic and classification purposes^41^ were externally validated. Eight were reported in journal articles^33^^,^^34^^,^^36^, ^37^, ^38^, ^39^, ^40^, ^41^ and 2 in conference proceedings.^32^^,^^35^ The datasets, preprocessing steps, and software used for annotation purposes are shown in Table 1.

**Table 1:** Datasets, sources, and preprocessing steps for development and validation of ML models and software used for annotation purposes.

	Diagnostic models			Classification *[continues]*	
Study	Cano et al. (2018)	Cruz-Roa et al. (2017)	Cruz-Roa et al. (2018)	Colon-Cartagena et al. (2020)	Mi et al. (2021)
Country of study	Colombia & United States	Colombia & United States	Colombia & United States	United States	China
Type of tumors/entities	Invasive ductal carcinomas	ER+ invasive breast cancer	ER+ invasive breast cancer	High-grade DCIS	Training/IV: Normal, benign, DCIS, and invasive carcinomas
					EV: Entities from BreakHis^a^ and BACH^b^
Training/tuning datasets: source (*n*)	HUP^c^ (239) TCGA^c^ (172)	HUP (239) CWRU/UHCMC (110)	HUP (239) CWRU (110)	VCU (334)	PUMCH (371)
IV datasets: source (n)	TCGA^c^ (172) HUP^c^ (239)	CINJ (40)	CINJ (40)	VCU (80)	PUMCH^d^ (169)
EV datasets: source (n)	CINJ (40)	TCGA (195) CWRU/UHCMC (21)^e^	TCGA (195)	NS (31)	BreakHis (7909) BACH (430)
Preprocessing steps	NS	Color normalization Data augmentation	Color normalization Data augmentation	NS	Data augmentation Image resizing Random color perturbations
Software used for annotation	NS	ImageScope v11.2 (Aperio) Image Viewer v3.1.4 (Ventana)	ImageScope v11.2 (Aperio) Image Viewer v3.1.4 (Ventana)	NS	ASAP

			
	Classification *[continued]*		Prognosis		Prognosis & Classification
Study	Radiya-Dixit et al. (2017)	Yang et al. (2019)	Bai et al. (2021)	Bychkov et al. (2022)	Wang et al. (2021)
Country of study	United States	China	Sweden & United States	Finland	Sweden
Type of tumors / entities	DCIS and UDH	Training/IV: Entities from BACH^b^	Triple-negative breast cancer	Breast cancer	Primary invasive breast cancer (types not specified)
		EV: Entities from BreakHis^a^			
Training / tuning datasets: source (*n*)	MGH (116)f	BACH (400)	Yale School of Medicine (95)^g^	FinProg (693)	SSGH, KUH, and TCGA (844)
IV datasets: source (n)	MGH (116) BIDMC (51)^f^	BACH (100)	Yale School of Medicine (171)^g^	FinProg (354)	SSGH, KUH, and TCGA (351)
EV datasets: source (n)	BIDMC (51)^f^	BreakHis (1995)	Yale School of Medicine (417)^g^ WTS Sweden (216) TCGA (116)	FinHer trial (712)	SCAN-B (1262)
Preprocessing steps	NS	Data augmentation	NS	Color normalization Data augmentation	Color normalization Data augmentation
Software used for annotation purposes	Fiji (ImageJ, National Institutes of Health)	NS	QuPath	WebMicroscope	NS

BIDMC: Beth Israel Deaconess Medical Center. CINJ: New Jersey Cancer Institute. CWRU: Case Western Reserve University. DCIS: Ductal Carcinoma in situ. ER+: Estrogen receptor-positive. EV: External validation. HUP: Hospital of the University of Pennsylvania. IV: Internal validation. KUH: Karolinska University Hospital. MGH: Massachusetts General Hospital. NS: Not specified. PUMCH: Peking Union Medical College Hospital. SCAN-B: Sweden Cancerome Analysis Network-Breast project. SSGH: Stockholm South General Hospital. TCGA: The Cancer Genome Atlas. UDH: Usual Ductal Hyperplasia. UHCMC: University Hospitals Case Medical Center. VCU: Virginia Commonwealth University.

a BreakHis dataset: (1) Benign lesions: Adenosis, Fibroadenomas, Phyllodes tumors, and Tubular adenoma. (2) Malignant tumors: Ductal carcinoma, Lobular carcinoma, Mucinous carcinoma, and Papillary carcinoma.

b BACH dataset: Normal, benign, carcinomas in situ, and invasive carcinomas.

c Internal validation with cases from the TCGA was conducted after the models were trained with cases from HUP, and internal validation with cases from HUP was conducted after the models were trained with cases from the TCGA.

d Internal validation dataset used by Mi et al. (2021) contained 115 paraffin-embedded tissues and 54 frozen section samples.

e External validation dataset used by Cruz-Roa et al. (2017) included positive and negative controls. Positive controls were extracted from the TCGA. Negative controls were extracted from normal breast tissue regions adjacent to invasive ductal carcinomas of patients diagnosed at UHCMC/CWRU.

f External validation performed with cases from the BIDMC after training the model with cases from the MGH. All cases from both institutions were combined to train and test the model when conducting the internal validation (using cross-validation).

g Training, IV, and EV datasets contained cases from different cohorts of patients of the Yale School of Medicine.

The reported information about the type of images, and the software platforms used for annotating and computational purposes and the type of tissues and tumors were largely heterogeneous among different studies. In those where relevant information was available, WSIs were employed^32^, ^33^, ^34^^,^^36^, ^37^, ^38^, ^39^, ^40^, ^41^ and only 2 studies included TMA images.^39^^,^^40^ Formalin-fixed, paraffin-embedded tissue samples were utilized in 3 studies,^37^^,^^40^^,^^41^ and frozen section samples were used in 1 (for the internal validation dataset, in addition to paraffin-embedded tissues).^36^ All tissues were stained with hematoxylin and eosin^32^, ^33^, ^34^^,^^36^, ^37^, ^38^, ^39^, ^40^, ^41^ and the most common scanning magnification was 40×.^33^^,^^34^^,^^36^^,^^38^^,^^41^ The patches sizes ranged from 50 × 50^32^ to 2048 × 1536 pixels.^36^ The *procedures* employed to *remove the* breast tissues (e.g., core needle biopsies or mastectomies) were not specified. However, Radiya et al. stated that scanned images of “breast biopsies” were used.^37^ Two authors reported the software platforms used for computational purposes. Bychkov et al. used PyTorch to implement deep learning architectures^40^ and Wang et al. used Keras (2.2.4) framework with TensorFlow (1.12) backend.^41^ Both used ADAM for optimization.^40^^,^^41^

Regarding the algorithms, 9 studies used convolutional neural networks (CNN). ResNet^35^^,^^38^^,^^40^ and DenseNet-161^38^ were used as backbone networks in three studies and InceptionV3 in 2.^36^^,^^41^ One study used a “Combined model with Active Feature Extraction” (CAFE), based on two logistic regression algorithms.^37^ A detailed description of each algorithm is shown in Appendix D.

### Risk of bias in studies

**Fig. 3:**
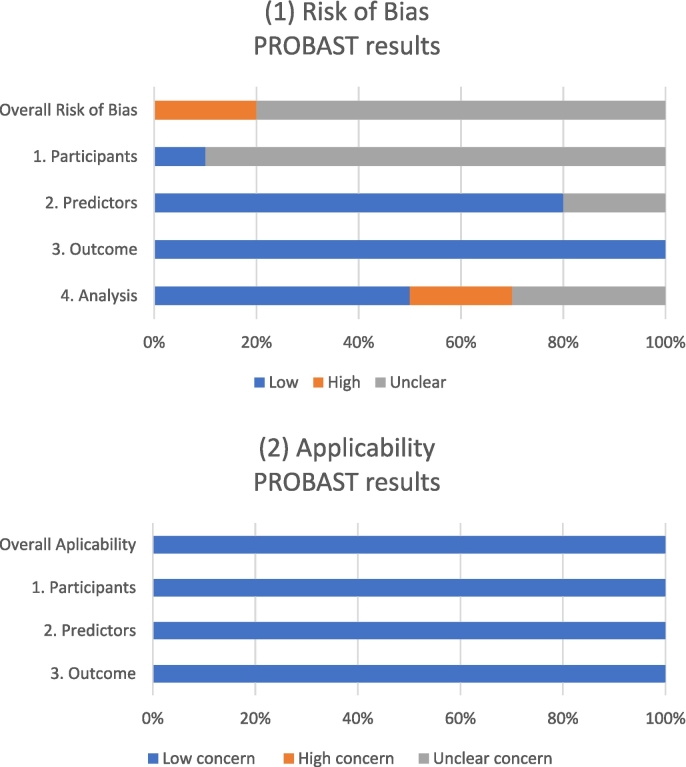
Prediction model Risk Of Bias Assessment Tool (PROBAST) Graphical presentation—(1) risk of bias results and (2) applicability.

According to the PROBAST,^42^ 2 studies were at a high risk of bias (ROB) in Domain 4 (Analysis) and the Overall judgment due to a small sample size of the validation dataset.^35^^,^^37^ For other studies, the ROB in Domain 4 (Analysis) and the Overall ROB was unclear due to the lack of more detailed information about the statistical analyses (studies validating prognostic ML models),^39^, ^40^, ^41^ the ROB in Domain 1 (Participants) and the Overall judgment was unclear due to the lack of more detailed information about the datasets (in all but one),^32^, ^33^, ^34^, ^35^, ^36^, ^37^, ^38^, ^39^^,^^41^ and the ROB in Domain 2 (Predictors) was unclear due to the lack of more detailed information on how predictors were defined or assessed.^35^^,^^37^ There was “low concern” regarding applicability for all studies (i.e., the population, predictors, or outcomes of the studies matched the systematic review question)^32^, ^33^, ^34^, ^35^, ^36^, ^37^, ^38^, ^39^, ^40^, ^41^ (Fig. 3).

### Results of individual studies

The ground truth used to assess the performance of models developed for diagnostic (i.e., to detect breast cancer) or classification (i.e., to separate different lesions of the breast) purposes were pathologists' annotations/opinions and, for prognostic models (i.e., to predict survival of patients), clinical data. The results of the external validation are summarized below. Related internal validation results are presented in Appendix E.

### ML for diagnostic purposes

While Cano et al. selected accuracy as the only performance metric for the EV of their model,^32^ Cruz Roa et al. reported several others.^33^^,^^34^ During the EV, their models were employed to detect the presence or absence of breast cancer, separating cases with and without invasive ductal carcinoma^32^ or with and without estrogen receptor-positive invasive breast cancer^33^^,^^34^ (as a binary classification problem) (Table 2).

**Table 2:** Results of EV of diagnostic models.

Study	Cano et al. (2018)	Cruz Roa et al. (2017)		Cruz-Roa et al. (2018)
Dataset		TCGA	CWRU/UHCMC^a^	
Accuracy (%)	88.78	–	–	–
Dice coefficient(%)	–	75.86	–	76
Positive-predictive value (%)	–	71.62	–	72
Negative-predictive value (%)	–	96.77	100	97
True-positive rate (%)	–	86.91	–	87
True-negative rate (%)	–	92.18	99.64	92
False-positive rate (%)	–	7.82	0.36	8
False-negative rate (%)	–	13.09	–	13
Heatmaps	–	–	–	√

CWRU: Case Western Reserve University. TCGA: The Cancer Genome Atlas. UHCMC: University Hospitals Case Medical Center. √: Good concordance between predictions of HASHI and pathologists' annotations.

a Not all performance metrics were calculated because the “normal” dataset did not have cancer annotations.

### ML for classification purposes

The most common performance metric used in their EV was accuracy,^35^^,^^36^^,^^38^ followed by area under the curve (AUC).^37^^,^^38^ Mi et al. reported the accuracies of their model with 4 different magnifications when differentiating benign lesions (Adenosis, Fibroadenomas, Phyllodes tumors, and Tubular adenoma) from malignant tumors (Ductal carcinoma, Lobular carcinoma, Mucinous carcinoma, and Papillary carcinoma) in the BreakHis dataset (as a binary classification problem), and when separating cases labeled as normal, benign, ductal carcinoma in situ (DCIS), and invasive carcinoma in the BACH dataset, part A (as a multi-label classification problem).^36^ Radiya-Dixit et al. reported the AUC of the ROC curve when their model was used to categorize breast lesions as either benign usual ductal hyperplasia or DCIS (a binary classification problem).^37^ Yang et al. included the performance metrics for the entire dataset (overall accuracy, AUC, precision, and recall) and for the benign lesions and malignant tumors categories of the BreakHis dataset (as binary classification problems).^38^ In summary, accuracy ranged from 87.2% (in the BACH dataset, part A)^36^ to 99.75% (in the BreakHis dataset),^38^ AUC from 91.8% (differentiating usual ductal hyperplasia from DCIS)^37^ to 99.99% (BreakHis dataset),^38^ and precision and recall from 99.20% to 100% (BreakHis dataset).^38^ (Table 3)

**Table 3:** Results of EV of classification models.

Study	Colon-Cartagena et al. (2020)	Mi et al. (2021)			Radiya-Dixit et al. (2017)	Yang et al. (2019)	
Dataset/category		BreakHis (Magnification)	BACH, part A	BACH, part B		Benign	Malignant
Accuracy (%)	90	96.7 (4×) 97.6 (10×) 95.0 (20×) 93.3 (40×)	87.2	–	–	99.75	99.75
Area under the curve (%)	–	–	–	–	91.8	99.99	99.99
Precision (%)	–	–	–	–	–	100	99.2
Recall (%)	–	–	–	–	–	99.64	100
Heatmaps	–	–	–	√	–	–	–

√: Good concordance between predictions of HASHI and pathologists' annotations.

### ML for prognostic purposes

Bai et al. reported that all the tumor-infiltrating lymphocytes (TILs) variables had a significant prognostic association with overall survival (*P*≤.01 for all comparisons). However, as shown in Table 4, the prognostic association of each TILs variable was different on each external validation dataset. In addition, the derived easTILs variable score had a good correlation with the pathologist-read sTILs in the WTS Sweden cohort (Spearman *r* coefficient=0.63, *P*<.0001).^39^ Bychkov et al. found that both “Solo” models (i.e., to predict the distant disease-free survival data only) and those trained in a multitask fashion (i.e., predicting estrogen receptor and HER2 status together with the distant disease-free survival data) significantly predicted distant disease-free survival^40^ (Table 4).

**Table 4:** Results of EV of prognostic models for predicting survival.

Study	Bai et al. (2021)				Bychkov et al. (2022)	
Dataset/Model	TMA Yale1	TMA Yale2	WTS TCGA	WTS Sweden	“Solo” model	Multitask model
	Hazard ratios^a^ (95% CI) *P*-value					
High eTILs%	0.64 (0.43–0.94) 0.025	0.43 (0.26–0.69) 0.0005	0.09 (0.01–0.70) 0.02	NS	–	–
High etTILS%	0.51 (0.32–0.81) 0.004	0.47 (0.28–0.77) 0.003	0.1 (0.01–0.80) 0.03	NS	–	–
High esTILs	0.48 (0.25–0.89) 0.02	0.42 (0.24–0.76) 0.004	NS	NS	–	–
High eaTILs (mm^2^)	0.48 (0.31–0.74) 0.0009	0.62 (0.37–1.01) 0.06	0.1 (0.01–0.76) 0.03	NS	–	–
High easTILs	0.65 (0.43–0.98) 0.04	0.78 (0.48–1.26) 0.31	NS	0.54 (0.31–0.92) 0.02	–	–
Predicted as “High risk”	–	–	–	–	1.8 (1.3–2.7) 0.002	1.7 (1.2–2.6) 0.003

	Spearman *r* coefficient *P*-value					
High easTILs	NS	NS	NS	0.63^b^ 0.0001	–	–

	Concordance index (“c-index”)					
Predicted risk score (CNN output) vs. actual time-to-event data	–	–	–	–	0.57	0.57

95% CI: 95% confidence interval. eaTILs (mm2): Density of TILs over tumor region. easTILs: Density of TILs over stroma area that mimics the international TIL working group variable as read by pathologists. eTILs%: Proportion of TILs over tumor cells. esTILs%: Proportion of TILs over stromal cells. etTILs%: Proportion of TILs over all detected cells. sTIL: Stromal TILs. TILs: Tumor-infiltrating lymphocytes.

a Outcomes predicted: Distant disease-free survival in patients with higher TILs scores (Bai et al. (2021)) and distant disease-free survival in patients predicted as “High risk” by ML models (by Bychkov et al. (2022)).

b Good correlation found when the CNN11-derived easTLs variable score was compared with the pathologist-read sTILs assessment.

### ML for classification and prognosis purposes

Wang et al. achieved an AUC of 0.907 (95% CI, 0.88–0.93, *P* = .930) when separating Nottingham Histological Grade 1 and Nottingham Histological Grade 3 invasive breast carcinomas and predicted recurrence-free survival rates between DeepGrade-classified Nottingham Histological Grade 1 and 3 patients that were similar to that of clinically assigned Nottingham Histological Grade 1 and 3 (defining recurrence as “locoregional or distant relapses, contralateral tumors or death”). It also provided a significant prognostic value for stratification of Nottingham Histological Grade 2 (*P* = .0045), and those predicted as high grade showed an increased risk for recurrence, with a HR of 1.91 (95% CI, 1.11–3.29, *P* = .019). Pathologists’ opinions were used as the ground truth.^41^

## Discussion

### Interpretation and implications of the results

To our knowledge, this is the first systematic review specifically focused on assessing externally validated ML models based on histopathology images for diagnosis, classification, prognosis, or treatment outcome prediction in female breast cancer.

Validating ML models is essential to ensure they will perform the task they were developed for.^14^ In an internal validation, an input dataset is split into parts; one is used to train and potentially fine-tune a ML model, and the other to test it. An EV uses independently derived datasets (i.e., external) to train and test ML models.^11^^,^^15^, ^16^, ^17^ As patterns learned from hidden variables of training datasets are not expected to improve ML models’ performance when tested with independent datasets, only EV is regarded as proof of generalizability.^11^^,^^15^^,^^18^

Although 2011 journal articles and conference proceedings were identified with our search queries, the majority of them did not report an EV of their ML models. This limitation has also been described for other ML models developed for medical purposes^16^^,^^17^^,^^22^, ^23^, ^24^, ^25^, ^26^, ^27^, ^28^^,^^43^, ^44^, ^45^ and for diagnostic or predictive purposes on breast cancer patients specifically.^46^, ^47^, ^48^ As previously stated, this may be related to the difficulty in finding appropriate external datasets,^47^^,^^48^ non-adherence to guidelines that promote EV^11^^,^^46^^,^^48^, ^49^, ^50^, ^51^ and lack of awareness of their importance.^48^ Particularly, DP is not yet widely adopted, and robust datasets with reliable labels and appropriate follow-up information have only become available only until recently.^20^^,^^52^, ^53^, ^54^, ^55^, ^56^ Also, as some hold sensitive information or belong to private companies that cannot or do not want to share their data, they have not been publicly available.^47^ This will possibly change with an increased awareness of DP applications and benefits and its widespread adoption in upcoming years.^55^^,^^57^^,^^58^

The reason why more of the included studies validated ML models for diagnosis or classification purposes is unknown. However, it may be partly explained by the fact that ML models used to predict biomarkers were excluded during the selection process. This could have limited the number of eligible studies used for prognosis and prevented the inclusion of any utilized for treatment-response prediction.

The commercial introduction of scanners that could generate high-resolution WSIs began in the 1990s.^59^^,^^60^ However, as described by Cooper et al., only during the past half-decade ML models have become a driving force for advancements in pathology.^60^ This work encompasses the time when this domain gained and sustained traction and, similarly, based on the records reviewed during the Title/Abstract screening, the number of publications has been progressively increasing in the last half-decade.

Limited access to large datasets to train ML models may explain why most studies were conducted in the United States/Colombia, the United States, or China.^61^ Although Colombia has not been traditionally considered a leader in artificial intelligence (AI)-related research output, in 3 studies, the first authors were affiliated with Colombian institutions and used datasets extracted from the United States.

There was significant heterogeneity in the studies regarding the amount and type of information reported. In addition, the performance metrics, datasets, ML models, image preprocessing, patch sizes, magnifications, and platforms used for annotating/computational purposes were highly variable. This finding corroborates the lack of standardization on the methodology and reported information described by Fell et al.^62^ and found in previous systematic reviews, such as those reported by Mazo et al. with studies using AI tools to predict breast cancer recurrence,^47^ by Gao et al. with ML-based breast cancer risk prediction models,^63^ by Corti et al. with AI algorithms for prediction of treatment outcomes in breast cancer,^46^ by Nagendran et al. with deep learning algorithms for medical imaging,^16^ and by Yu et al. with deep learning algorithms with EV for radiologic diagnosis.^48^ As explained by other authors, this is a noteworthy limitation of these systematic reviews^48^ that impedes from making rigorous comparisons,^63^ better understand findings,^64^ and limits models' generalizability and their clinical impact.^46^ Increasing adherence to existing and upcoming reporting guidelines^11^^,^^26^^,^^49^^,^^50^^,^^65^^,^^66^ could be potentially improved by training authors on their practical use, enhancing the understanding or their content, encouraging and checking the adherence to them, and involving experts on methodology and reporting on AI research groups.^67^

It is recommended^14^ to test ML models with diverse and large datasets that address the variability found in real-world data and allow statistically meaningful analysis. Nevertheless, it was impossible to determine if the included studies were aligned with this recommendation. That is because detailed descriptions of the variability of the datasets were not included in the reports. In addition, as stated above, important differences among the studies in terms of the source, size, and histological type of tumors/entities included in the training/tuning and validating datasets were noted.

The majority of experiments were performed using WSIs. As for many other tools developed for computational pathology,^68^ most authors utilized images scanned with 20× and 40× magnifications. Data augmentation and color normalization were the most common preprocessing methods. Both have been widely used in computational pathology to help improve the generalizability of ML models. The first aims to increase the diversity of the training data by adding artificially generated variations of them (e.g., by rotating or mirroring the images).^69^ The latter tries to reduce the effect of color variations in the images (usually a consequence of different staining or scanning processes).^69^^,^^70^ Except for some similarities found in the 2 studies written by the same author,^33^^,^^34^ the software platforms used for annotation/computational purposes and the information published about them were different in each study.

All the included studies, but one, used CNNs, a category of deep neural networks (DNNs). Unlike traditional computer vision approaches that require designing hand-engineered features (usually time-consuming and expensive), DNNs can learn to extract features automatically.^71^ In addition this advantage, CNNs use filters (AKA convolutional kernels) that have shown to be very powerful in learning patterns from images and videos.^71^ Consequently, CNNs became dominant during the last years.^72^^,^^73^ Even though vision transformers have outperformed CNNs in some computer vision tasks, these started to be used more recently and were not found in any of the included studies.^20^^,^^74^

Considering that the performance of ML models when validated on external datasets usually diminishes, the results of most included studies can be regarded as encouraging. For example, all the accuracies and AUC achieved by the diagnostic or classification models were above 87% and 90%, respectively; Yang et al. obtained perfect or almost perfect precision and recall values, the prognostic models developed by Bychkov et al. and Wang et al., the hazard ratios (HR) were between 1.7 and 1.9 (with statistical significance), and all the machine TIL variables developed by Bai et al. were significantly associated with outcomes. However, as discussed below, the limited number of histological categories, classes, or types of tumors/entities in which the models were applied could restrict their usability in real-life clinical practices.

In the 2 studies where enough information was available, the risk of bias was high due to the small sample size of the validation dataset. Therefore, their predictive performance will likely be lower than that reported. Although PROBAST^42^ was originally created for statistical (regression-based) prediction models, most of its items are applicable to ML-based prediction model studies.^75^ As ML models need large sample sizes, insufficient sample sizes when developing and validating ML models can be considered a major design flaw.^75^

Although obtaining good results in one or more EV datasets is important evidence of generalizability,^15^ it is essential to note that this doesn't guarantee that the model will perform well in all other settings.^14^^,^^76^ As the amount and diversity of examples used to train ML models limit their prediction/recognition capabilities, only patterns properly represented in training datasets are expected to be adequately predicted/recognized in validation datasets.^69^^,^^76^, ^77^, ^78^ According to the Food and Drug Administration (FDA) and College of American Pathologists (CAP), validation datasets must contain sufficient cases representative of those the product/algorithm will likely encounter during its intended use.^79^^,^^80^ Thus, validation datasets may need to be constructed for each institution where ML models will be deployed.^14^^,^^76^ Also, as they may recurrently encounter new cases with previously unseen relevant attributes, monitoring ML models’ performance, and updating them (by retraining/fine-tuning their hyperparameters) recurrently could be necessary.^14^^,^^81^, ^82^, ^83^

**Fig. 4:**
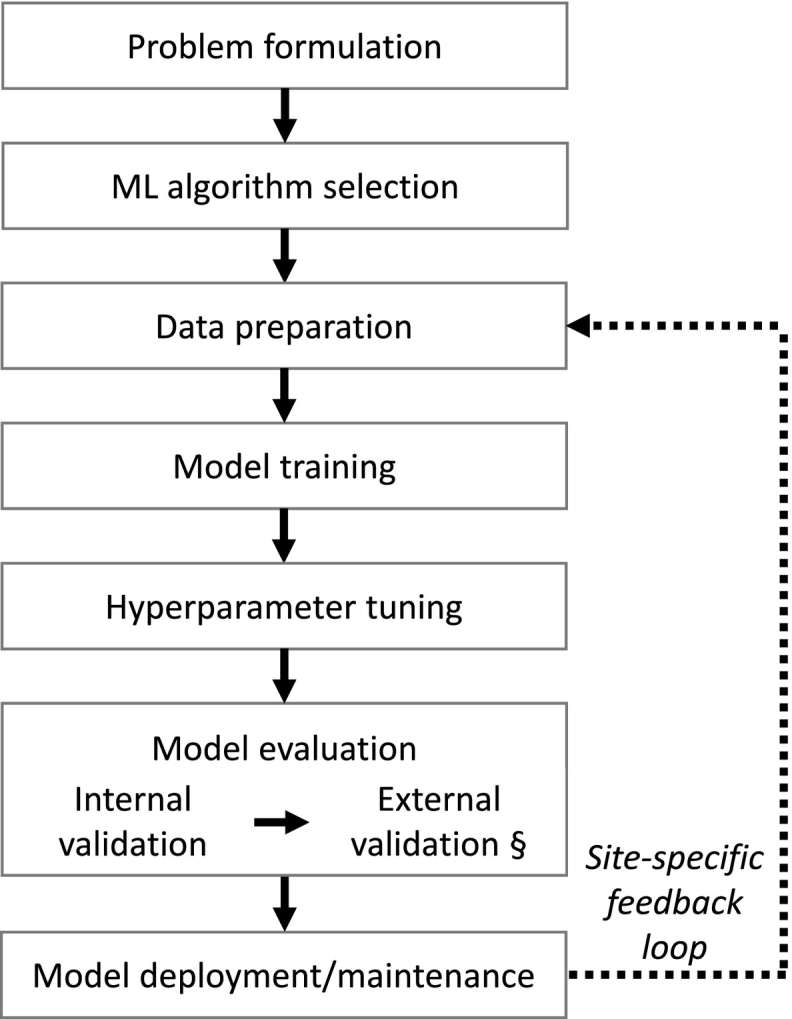
ML models site-specific iterative development steps review.

This can become an iterative process for each institution as shown in Fig. 4.

Gathering experiences prospectively could support the future development of guidelines that define some specifics around this workflow, such as the best way to measure whether retraining of the model is needed, potential thresholds of internal/external validations, the necessity of assessing diagnostically the incorrectly predicted EV data to ensure they have representation in the training datasets and the need of pathologists’ intervention to ensure that predictions are aligned with their diagnoses.

Also, considering that ML models may become more generalizable if retrained/fine-tuned with diverse data extracted from the institutions that use them, the implications of this improvement could be discussed between these institutions and vendors when models are privately owned.^84^

### Limitations of the review process and of the evidence included in the review

Similar to what has been described by several authors before,^46^, ^47^, ^48^^,^^63^ there is an observed lack of consistency in the methods, performance metrics of the studies explored in this work. Additionally, the EVs utilized for each of the studies are heterogeneous and therefore limit the comparison across all the works and possibly inhibit a deeper understanding of how the results compare.

It is also relevant to mention that all the studies that externally validated ML models for diagnosis or classification purposes, trained, and validated the models with broad categories (or classes) of tumors/entities or with a few specific subset of them (e.g., only some histological types or subtypes). In clinical practices, pathologists always need to be able to recognize all the specific types and subtypes of tumors/entities listed in the classifications regarded as standard. The World Health Organization (WHO) classifications are the most commonly used classifications for human tumors.^85^ And as an example, the class “invasive breast carcinoma” contains more than 20 specific histological types of tumors in its most recent edition (and some of them can be further subclassified in subtypes and variants).^86^ Although training/testing a model to recognize all the specific types and subtypes of tumors/entities could be out of the scope of all or almost all ML models, this limitation will also undoubtedly restrict their applicability in clinical settings.

Although the search strategies were designed to be very comprehensive (See Appendix B), the studies that did not suggest, imply, or mention that their ML models underwent external validation in their Title/Abstract were not included. The same happened with those published (and indexed) in MEDLINE, EMBASE, CINAHL, IEEE, MICCAI, or SPIE conferences before January 1, 2010, or after February 28, 2022. Consequently, this systematic review did not include some ML models that might have otherwise met the inclusion criteria. For example, a study showing the results of the external validation of GALEN Breast, a commercially available algorithm used to detect invasive and in situ breast carcinomas, was excluded because it was published in December 2022.^87^ Besides, numerous ML models not developed for diagnosis, classification, prognosis, or treatment outcome prediction (such as some recently reviewed by Chan et al.),^88^ even if they became commercially available and were externally validated, were excluded for not meeting the inclusion criteria (e.g., had a different purpose).

Future comprehensive analyses may be facilitated with an increased availability of external validation datasets and by enhancing adherence to standardized methods and reporting protocols.

## Registration and protocol

The review was not registered.

## Declaration of Competing Interest

Authors do not have any competing interests to declare. This research did not receive any specific grant from funding agencies in the public, commercial, or not-for-profit sectors.

## Figures and Tables

**Fig. 5:**
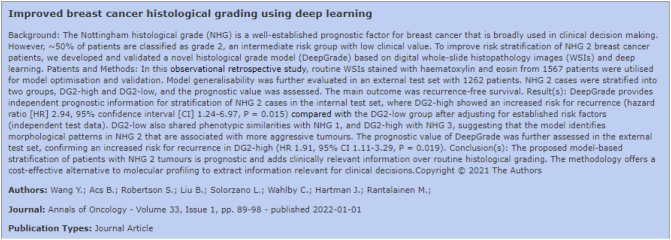
Record that passed the Title/Abstract screening and the full-text screening.

**Fig. 6:**
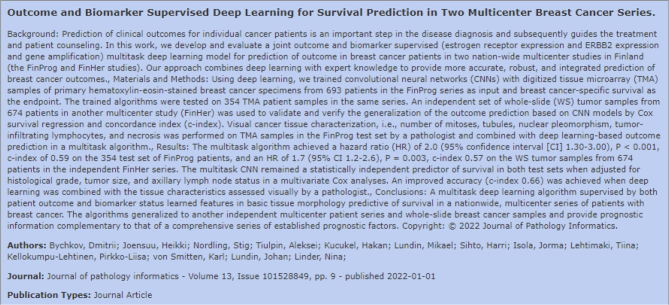
Record that passed the Title/Abstract screening and the full-text screening.

**Fig. 7:**
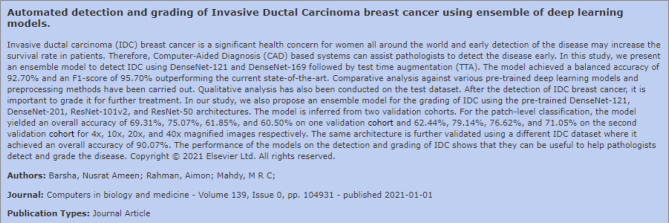
Record that passed the Title/Abstract screening but did not pass the full-text screening (because EV was not performed).

**Fig. 8:**
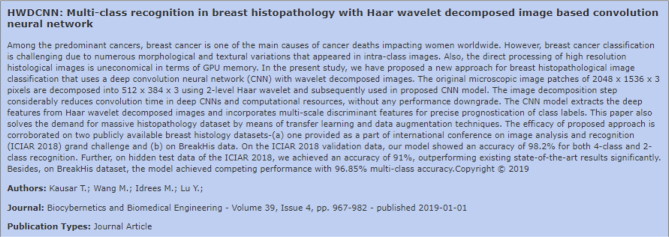
Record that passed the Title/Abstract screening but did not pass the full-text screening (because EV was not performed).

**Fig. 9:**
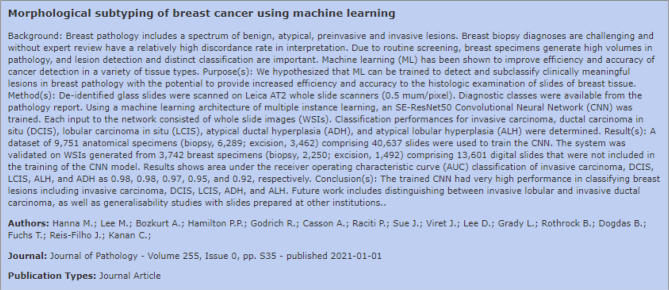
Record that did not pass the Title/Abstract screening (because EV was not performed).

**Fig. 10:**
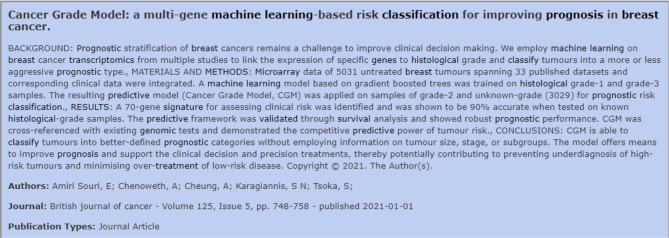
Record that did not pass the Title/Abstract screening (because the model was only trained with microarray and clinical data).

**Fig. 11:**
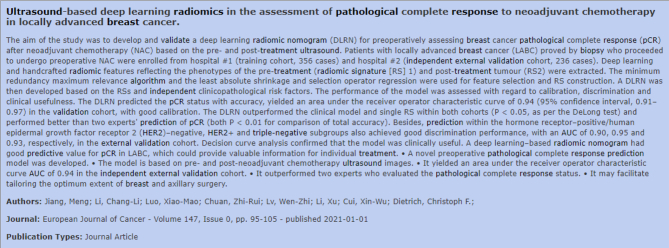
Record that did not pass the Title/Abstract screening (because the model was only trained with ultrasound images).

**Fig. 12:**
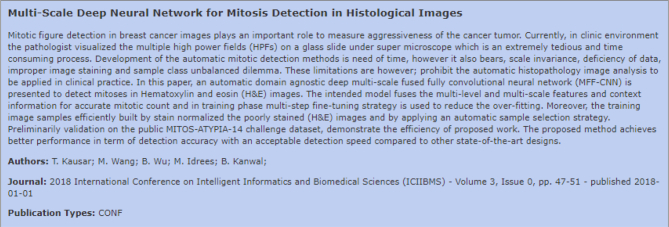
Record that did not pass the Title/Abstract screening (because the model was developed to detect mitoses).

**Fig. 13:**
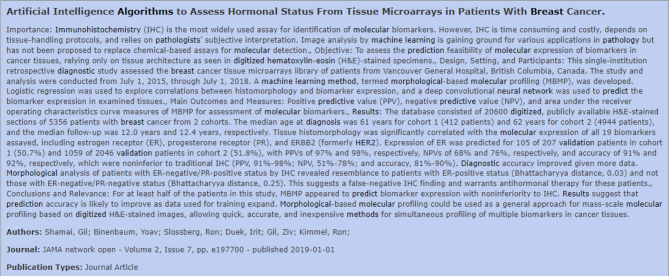
Record that did not pass the Title/Abstract screening (because the model was developed to predict the expression of biomarkers).

**Table:** 

Authors	Algorithms
Cano et al.	IDC-CNN 9L-16n: 9 layers: 3 convolutional-pooling layers of 16 neurons per layer, 3 convolutional-pooling layers of 32 neurons per layer, 1 fully connected layer of 16 neurons, 1 fully connected layer of 32 neurons, and 1 sigmoid classification layer
Cruz-Roa et al.	A 3-layer CNN. One convolutional and pooling layer and a fully connected layer (each with 256 neurons). The third one was a classification layer, with 2 units as outputs (one for each class; invasive and non-invasive)
Cruz-Roa et al.	CS256-FC256: One convolutional and subsampling/pooling layer (with 256 neurons), a fully connected layer (with 256 neurons), and a final classification layer (Softmax classifier)
Colon-Cartagena et al.	- ResNet-50 model - A coding-free custom image classifier and visual recognition model (created with the IBM Watson Visual Recognition platform)
Mi et al	Inception V3 was selected as the patch-level classifier. A full connection layer (with 1024 neurons) and a Softmax layer were added after the basic model
Radiya-Dixit et al	Best performance model: Combined model with Active Feature Extraction - CAFE; consisting of two logistic regression algorithms
Yang et al	Nine fine-tuned deep CNN were obtained using ResNet-101, ResNet-152, and DenseNet-161 as backbone networks. The combination of the 3 of them achieved the best performance
Bai et al	CNN11 TIL algorithm (Neural network with 8 hidden layers)
Bychkov et al.	Deep learning model around a ResNet CNN backbone (with a dropout layer introduced before the fully connected blocks)
Wang et al.	InceptionV3 (with weights initialized from a model pretrained by ImageNet)

**Table 5:** Results of internal validation of diagnostic models.

Study	Cano et al. (2018)	Cruz Roa et al. (2017)		Cruz-Roa et al. (2018)
Dataset		ConvNet_HUP_	ConvNet_UHCMC/CWRU_	
Accuracy (%)	90.07	–	–	–
Dice coefficient(%)	–	67.71	65.96	67
Positive-predictive value (%)	–	64.64	63.70	–
Negative-predictive value (%)	–	97.09	96.63	–
True-positive rate (%)	–	–	–	–
True-negative rate (%)	–	–	–	–
False-positive rate (%)	–	–	–	–
False-negative rate (%)	–	–	–	–
Heatmaps	–	–	–	–

ConvNet_HUP_: Convolutional Neural Network trained with cases from the Hospital of the University of Pennsylvania. ConvNet_UHCMC/CWRU_: Convolutional Neural Network trained with cases from the University Hospitals Case Medical Center and Case Western Reserve University.

**Table 6:** Results of internal validation of classification models.

Study	Colon-Cartagena et al. (2020)	Mi et al. (2021)				Radiya-Dixit et al. (2017)	Yang et al. (2019)				
Dataset/category		Normal	Benign	In situ	Invasive		Normal	Benign	In situ	Invasive	All
Accuracy (%)	90	86.67				–	95.98	94.24	96.01	97.26	91.75
Area under the curve (%)	–	97.56	96.62	98.37	95.15	92.1	98.78	96.40	98.38	98.50	–
Precision (%)	–	80.77	80.95	94.41	92.88	–	89.27	97.65	91.36	92.57	–
Recall (%)	–	92.75	83.12	87.78	82.75	–	97	79	94	97	–
Heatmaps	–	–	–	–	–	–	–	–	–	–	–

**Table 7:** Results of internal validation of prognostic models.

Study	Bai et al. (2021)	Bychkov et al. (2022)	
Dataset/Model	WTS Yale	“Solo” model	Multitask model
	Hazard ratios^a^ (95% CI) *P*-value		
High eTILs%	0.35 (0.20–0.61) 0.0002	–	–
High etTILS%	0.35 (0.19–0.63) 0.0005	–	–
High esTILs	0.35 (0.18–0.65) 0.001	–	–
High eaTILs (mm2)	0.35 (0.20–0.63) 0.0005	–	–
High easTILs	0.30 (0.16-0.54) <0.0001	–	–
Predicted as “High risk”	–	1.7 (1.1-2.6) ≤0.01	2 (1.3-3.0) ≤0.001

	Spearman *r* coefficient *P*-value		
High easTILs	0.61^b^ 0.0001	–	–

	Concordance index (“c-index”)		
Predicted risk score (CNN output) vs. actual time-to-event data	–	0.57	0.59

95% CI: 95% confidence interval. eaTILs (mm2): Density of TILs over tumor region. easTILs: Density of TILs over stroma area that mimics the international TIL working group variable as read by pathologists. eTILs%: Proportion of TILs over tumor cells. esTILs%: Proportion of TILs over stromal cells. etTILs%: Proportion of TILs over all detected cells. sTIL: Stromal TILs. TILs: Tumor-infiltrating lymphocytes.

a Outcomes predicted: Distant disease-free survival in patients with higher TILs scores (Bai et al. (2021)) and distant disease-free survival in patients predicted as “High risk” by ML models (by Bychkov et al. (2022)).

b Good correlation found when the CNN11-derived easTLs variable score was compared with the pathologist-read sTILs assessment.

## Data Availability

The data that support the findings of this study are available on request from the corresponding author.

## Appendix A Databases description

### A.1 Ovid MEDLINE®

Produced by the National Library of Medicine (NLM). It indexes information from approximately 5600 journals published world-wide, related to “biomedicine, including the allied health fields and the biological and physical sciences, humanities, and information science as they relate to medicine and health care”.^89^ Ovid MEDLINE® also contains PubMed-not-MEDLINE records from NLM^89^ (i.e., journals/manuscripts deposited in PubMed Central and books and documents from the National Center for Biotechnology Information (NCBI) Bookshelf).^90^

### A.2 Embase (Excerpta Medica Database)

Produced by Elsevier B.V. Indexes information from more than 8500 journals published world-wide biomedicine, and “is especially strong in its coverage of drug and pharmaceutical research, pharmacology, toxicology, and medical devices.”^91^

### A.3 CINAHL

Produced by EBSCO. Indexes the top nursing and allied health literature available from more than 3800 journals.^92^

### A.4 IEEE Xplore

Produced by the Institute of Electrical and Electronics Engineers (IEEE), Indexes more than 6 million full-text documents (e.g., journals, conference papers, technical standards, and books) of electrical engineering, computer science, and electronics.^93^

### A.5 MICCAI

The proceedings of the International Conference on Medical Image Computing and Computer-Assisted Intervention (MICCAI) are published in the Springer Lecture Notes in Computer Science (LNCS) series.^94^ It includes oral and poster papers on “medical image computing, computer-assisted intervention, guidance systems and robotics, visualization and virtual reality, computer-aided diagnosis, bioscience and biology applications, specific imaging systems, and new imaging applications”.^95^

### A.6 SPIE conferences

Each year the Society of Photo-Optical Instrumentation Engineers (SPIE) conferences indexes 16 000+ papers and presentation recordings “reporting on photonics-driven advancements in biomedicine, astronomy, defense and security, renewable energy”^96^

## Appendix B Search strategies

### B.1 OVID Medline Epub Ahead of Print, In-Process & Other Non-Indexed Citations, Ovid MEDLINE(R) Daily, and Ovid MEDLINE(R) 1946 to Present

1. “neoplasms, glandular and epithelial”/ or carcinoma/ or adenocarcinoma, mucinous/ or carcinoma, adenoid cystic/ or carcinoma, ductal/ or carcinoma, ductal, breast/ or carcinoma, lobular/ or carcinoma, mucoepidermoid/ or carcinoma, neuroendocrine/ or cystadenocarcinoma, mucinous/ or carcinoma, adenosquamous/ or carcinoma, papillary/ or carcinoma, squamous cell/ or “neoplasms, cystic, mucinous, and serous”/ or cystadenocarcinoma/ or “neoplasms, ductal, lobular, and medullary”/ or carcinoma, medullary/ or breast neoplasms/ or “hereditary breast and ovarian cancer syndrome”/ or inflammatory breast neoplasms/ or triple negative breast neoplasms/ or unilateral breast neoplasms/

2. (cancer or carcinoma$ or malignant).tw.

3. breast/ or mammary glands, human/

4. (breast or mammar$).tw.

5. 3 or 4

6. 2 and 5

7. 1 or 6

8. algorithms/ or artificial intelligence/ or machine learning/ or deep learning/ or supervised machine learning/ or support vector machine/ or unsupervised machine learning/ or decision theory/ or decision trees/ or neural networks, computer/

9. machine learning.tw. or exp algorithm/ or algorithm.tw. or Automatic Data Processing/ or Automatic Data Processing.tw. or computer aided detection.tw. or artificial intelligence.tw.

10. 8 or 9

11. pathology/ or pathology, surgical/ or histology/ or histocytochemistry/ or immunohistochemistry/

12. (histopatholog$ or patholog$ or histolog$).tw.

13. (“whole slide imag$” or WSI or (digit$ and slide$)).tw.

14. 11 or 12 or 13

15. valid*.tw.

16. diagnosis/ or clinical decision-making/ or clinical reasoning/ or diagnosis, computer-assisted/ or image interpretation, computer-assisted/ or diagnosis, differential/ or specimen handling/ or biopsy/ or biopsy, needle/ or biopsy, large-core needle/ or dissection/ or microscopy/ or photomicrography/ or diagnostic tests, routine/ or early diagnosis/ or “early detection of cancer”/ or prognosis/ or neoplasm staging/ or treatment outcome/ or disease-free survival/ or progression-free survival/ or response evaluation criteria in solid tumors/ or treatment failure/

17. (diagnos$ or classif$ or prognos$ or predict$).tw.

18. 16 or 17.

### B.2 Embase 1974 to 2022 February 28

1. neoplasm/ or malignant neoplasm/ or solid malignant neoplasm/ or carcinoma/ or breast tumor/ or breast cancer/ or breast carcinoma/ or breast adenocarcinoma/ or metastatic breast cancer/ or ductal carcinoma/ or breast ductal carcinoma/ or lobular carcinoma/ or inflammatory breast cancer/ or metaplastic carcinoma/ or adenosquamous carcinoma/ or papillary carcinoma/ or medullary carcinoma/ or neuroendocrine carcinoma/ or adenoid cystic carcinoma/ or breast cancer molecular subtype/ or luminal A breast cancer/ or luminal B breast cancer/ or basal like breast cancer/ or triple negative breast cancer/ or estrogen receptor positive breast cancer/ or estrogen receptor negative breast cancer/ or human epidermal growth factor receptor 2 positive breast cancer/ or human epidermal growth factor receptor 2 negative breast cancer/ or “hereditary breast and ovarian cancer syndrome”/

2. (cancer or carcinoma$ or malignant).tw.

3. breast/ or mammary gland/

4. (breast or mammar$).tw.

5. 3 or 4

6. 2 and 5

7. 1 or 6

8. machine learning/ or artificial neural network/ or computer vision/ or automated pattern recognition/ or back propagation/ or bayesian learning/ or classification algorithm/ or classifier/ or clustering algorithm/ or computer heuristics/ or convolution algorithm/ or cross validation/ or data mining/ or deconvolution algorithm/ or detection algorithm/ or Dijkstra's algorithm/ or dimensionality reduction/ or dynamic time warping/ or empirical mode decomposition/ or feature detection/ or feature extraction/ or feature extraction algorithm/ or “feature learning (machine learning)”/ or feature ranking/ or feature selection/ or feature selection algorithm/ or fuzzy system/ or generalized method of moments/ or greedy algorithm/ or hidden markov model/ or imaging algorithm/ or iterative closest point/ or k nearest neighbor/ or kernel method/ or knowledge discovery/ or learning algorithm/ or Levenberg Marquardt algorithm/ or Markov jump/ or markov state model/ or maximum entropy model/ or maximum likelihood method/ or Metropolis Hastings algorithm/ or model predictive control/ or multicriteria decision analysis/ or multifactor dimensionality reduction/ or Needleman Wunsch algorithm/ or network learning/ or online analytical processing/ or perceptron/ or radial basis function/ or radial basis function/ or random forest/ or recursive feature elimination/ or recursive partitioning/ or relevance vector machine/ or risk algorithm/ or rough set/ or successive projections algorithm/ or semi supervised machine learning/ or superposition algorithm/ or supervised machine learning/ or support vector machine/ or unsupervised machine learning/

9. machine learning.mp. or machine learning/ or algorithm.mp. or algorithm/ or information processing.mp. or information processing/ or artificial intelligence.mp. or artificial intelligence/

10. 8 or 9

11. pathology/ or general pathology/ or histopathology/ or molecular pathology/ or pathological anatomy/ or neuropathology/ or histology/ or histometry/ or histophotometry/ or brain histology/ or liver histology/ or immunohistochemistry.mp. [mp=title, abstract, heading word, drug trade name, original title, device manufacturer, drug manufacturer, device trade name, keyword heading word, floating subheading word, candidate term word]

12. (histopatholog$ or patholog$ or histolog$).tw.

13. (“whole slide imag$” or WSI or (digit$ and slide$)).tw.

14. 11 or 12 or 13

15. valid$.tw.

16. diagnosis/ or early diagnosis/ or tumor diagnosis/ or cancer diagnosis/ or early cancer diagnosis/ or differential diagnosis/ or diagnosis related group/ or tumor biopsy/ or diagnostic test/ or cancer test/ or laboratory diagnosis/ or molecular diagnosis/ or computer assisted diagnosis/ or quantitative diagnosis/ or diagnostic accuracy/ or diagnostic test accuracy study/ or diagnostic error/ or missed diagnosis/ or diagnostic reasoning/ or cancer grading/ or cancer staging/ or prognosis/ or cancer prognosis/ or disease course/ or survival/ or survival prediction/ or cancer survival/ or cancer specific survival/ or survival rate/ or mean survival time/ or median survival time/ or overall survival/ or survival analysis/ or long term survival/ or cancer free survival/ or disease free survival/ or disease specific survival/ or disease free interval/ or event free survival/ or progression free survival/ or recurrence free survival/ or local progression free survival/ or distant progression free survival/ or local recurrence free survival/ or distant recurrence free survival/ or local disease free survival/ or distant disease free survival/ or metastasis free survival/ or distant metastasis free survival/ or failure free survival/ or local failure free survival/ or regional failure free survival/ or distant failure free survival/ or treatment free survival/ or post treatment survival/ or cancer recurrence/ or cancer regression/ or tumor recurrence/ or tumor regression/ or recurrent disease/ or relapse/ or remission/ or prediction/ or computer prediction/ or forecasting/ or predictive validity/ or predictive value/ or outcome assessment/ or response evaluation criteria in solid tumors/ or treatment response/ or treatment response time/ or treatment failure/ or treatment outcome/ or disease worsening with drug treatment/ or outcomes research/

17. (diagnos$ or classif$ or prognos$ or predict$).tw.

18. 16 or 17

### B.3 CINAHL. Interface: EBSCOhost Research Databases

( ((MH “Neoplasms, Glandular and Epithelial”) OR (MH “Carcinoma”) OR (MH “Carcinoma, Adenoid Cystic”) OR (MH “Carcinoma, Ductal”) OR (MH “Carcinoma, Ductal, Breast”) OR (MH “Carcinoma, Lobular”) OR (MH “Carcinoma, Neuroendocrine”) OR (MH “Carcinoma, Papillary”) OR (MH “Carcinoma, Squamous Cell”) OR (MH “Neoplasms, Cystic, Mucinous, and Serous”) OR (MH “Neoplasms, Ductal, Lobular, and Medullary”) OR (MH “Breast Neoplasms”) OR (MH “Hereditary Breast and Ovarian Cancer Syndrome”) OR (((MH “Breast”) OR (TI breast OR AB breast) OR (TI mammar* OR AB mammar*)) AND ((TI cancer OR AB cancer) OR (TI carcinoma* OR AB carcinoma*) OR (TI malignant OR AB malignant)))) ) AND ( ((MH “Algorithms”) OR (MH “Artificial Intelligence”) OR (MH “Machine Learning”) OR (MH “Deep Learning”) OR (MH “Support Vector Machine”) OR (MH “Decision Trees”) OR (MH “Neural Networks (Computer)”) OR (TI “machine learning” OR AB “machine learning”) OR (TI algorithm OR AB algorithm) OR (TI “Automatic Data Processing” OR AB “Automatic Data Processing”) OR (TI “computer aided detection” OR AB “computer aided detection”) OR (TI “artificial intelligence“ OR AB “artificial intelligence”)) ) AND ( ((MH “Pathology”) OR (MH “Histology”) OR (MH “Histocytochemistry”) OR (MH “Immunohistochemistry”) OR (TI histopathology* OR AB histopathology*) OR (TI patholog* OR AB patholog*) OR (TI histolog* OR AB histolog*) or ("whole slide imag*" OR WSI OR (digit* AND slide*))) ) AND ( (TI valid* OR AB valid*) ) OR ( ((MH “Diagnosis”) OR (MH “Decision Making, Clinical”) OR (MH “Clinical Reasoning”) OR (MH “Diagnosis, Computer Assisted”) OR (MH “Image Interpretation, Computer Assisted”) OR (MH “Diagnosis, Differential”) OR (MH “Specimen Handling”) OR (MH “Biopsy”) OR (MH “Biopsy, Needle”) OR (MH “Dissection”) OR (MH “Microscopy”) OR (MH “Diagnostic Tests, Routine”) OR (MH “Early Diagnosis”) OR (MH “Early Detection of Cancer”) OR (MH “Prognosis”) OR (MH “Neoplasm Staging”) OR (MH “Treatment Outcomes”) OR (MH “Treatment Failure”) OR (TI diagnos* OR AB diagnos*) OR (TI diagnos* OR AB diagnos*) OR (TI classif* OR AB classif*) OR (TI prognos* OR AB prognos*) or (TI predict* OR AB predict*)) ).

### B.4 IEEE via IEEE *Xplore*

((“Mesh_Terms”:“neoplasms, glandular and epithelial” OR “Mesh_Terms”:carcinoma OR “Mesh_Terms”:adenocarcinoma, mucinous OR “Mesh_Terms”:carcinoma, adenoid cystic OR “Mesh_Terms”:carcinoma, ductal OR “Mesh_Terms”:carcinoma, ductal, breast OR “Mesh_Terms”:carcinoma, lobular OR “Mesh_Terms”:carcinoma, mucoepidermoid OR “Mesh_Terms”:carcinoma, neuroendocrine OR “Mesh_Terms”:cystadenocarcinoma, mucinous OR “Mesh_Terms”:carcinoma, adenosquamous OR “Mesh_Terms”:carcinoma, papillary OR “Mesh_Terms”:carcinoma, squamous cell OR “Mesh_Terms”:“neoplasms, cystic, mucinous, and serous” OR “Mesh_Terms”:cystadenocarcinoma OR “Mesh_Terms”:“neoplasms, ductal, lobular, and medullary” OR “Mesh_Terms”:carcinoma, medullary OR “Mesh_Terms”:breast neoplasms OR “Mesh_Terms”:“hereditary breast and ovarian cancer syndrome” OR “Mesh_Terms”:inflammatory breast neoplasms OR “Mesh_Terms”:triple negative breast neoplasms OR “Mesh_Terms”:unilateral breast neoplasms) OR (((“Index Terms”:cancer OR “Index Terms”:carcinoma OR “Index Terms”:malignant) OR (“Abstract”:cancer OR “Abstract”:carcinoma OR “Abstract”:malignant)) AND ((“Mesh_Terms”:breast OR “Mesh_Terms”:mammary glands, human) OR (“Index Terms”:breast OR “Index Terms”:mammary OR “Index Terms”:mammarian) OR (“Abstract”:breast OR “Abstract”:mammary OR “Abstract”:mammarian)))) AND ((“Mesh_Terms”:algorithms OR “Mesh_Terms”:artificial intelligence OR “Mesh_Terms”:machine learning OR “Mesh_Terms”:deep learning OR “Mesh_Terms”:supervised machine learning OR “Mesh_Terms”:support vector machine OR “Mesh_Terms”:unsupervised machine learning OR “Mesh_Terms”:decision theory OR “Mesh_Terms”:decision trees OR “Mesh_Terms”:neural networks, computer) OR ("IEEE Terms":Machine learning algorithms OR “IEEE Terms”:Artificial intelligence OR “IEEE Terms”:Machine learning OR “IEEE Terms”:Deep learning OR “IEEE Terms”:Supervised learning OR “IEEE Terms”:Unsupervised learning OR “IEEE Terms”:Semisupervised learning OR “IEEE Terms”:Support vector machines OR “IEEE Terms”:Artificial neural networks) OR (“Index Terms”:machine learning OR “Index Terms”:artificial intelligence) OR (“Abstract”:machine learning)) AND ((“Mesh_Terms”:pathology OR “Mesh_Terms”:pathology, surgical OR “Mesh_Terms”:histology OR “Mesh_Terms”:histocytochemistry OR “Mesh_Terms”:immunohistochemistry) OR (“Index Terms”:histopathology OR “Index Terms”:histopathological OR “Index Terms”:pathology OR “Index Terms”:pathological OR “Index Terms”:histology OR “Index Terms”:histological) OR (“Abstract”:histopathology OR “Abstract”:histopathological OR “Abstract”:pathology OR “Abstract”:pathological OR “Abstract”:histology OR “Abstract”:histological) OR (“Index Terms”:“whole slide images” OR “Index Terms”:“whole slide imaging” OR “Index Terms”:WSIs) OR (“Abstract”:“whole slide images” OR “Abstract”:“whole slide imaging” OR “Abstract”:WSIs) OR ((“Index Terms”:digital OR “Index Terms”:digitizing OR “Index Terms”:digitized) AND (“Index Terms”:slides)) OR ((“Abstract”:digital OR “Abstract”:digitizing OR “Abstract”:digitized) AND (“Abstract”:slides))) AND ((“Index Terms”:valid*) OR (“Abstract”:valid*)).

### B.5 MICCAI via Springer link

(Breast OR mammary) AND (Cancer OR carcinoma OR tumor OR tumors OR tumour OR tumours OR neoplasm OR neoplasms OR malignant) AND (“artificial intelligence” OR “machine Learning” OR “Deep learning” OR “neural network” OR “computer vision” OR Algorithm OR Algorithms) AND (pathology OR histology OR histopathology OR histological OR histopathological OR “whole slide images” OR “whole slide imaging” OR WSI OR WSIs OR “digital slides”) AND (valid*). Filters: within miccai & Conference Paper & 2010–2022.

### B.6 SPIE conferences

ABSTRACT:(Breast OR mammary) AND ABSTRACT:(Cancer OR carcinoma OR tumor OR tumors OR tumour OR tumours OR neoplasm OR neoplasms OR malignant) AND ABSTRACT:(“artificial intelligence” OR “machine Learning” OR “Deep learning” OR “neural network” OR “computer vision” OR Algorithm OR Algorithms) AND ABSTRACT:(pathology OR histology OR histopathology OR histological OR histopathological OR “whole slide images” OR “whole slide imaging” OR WSI OR WSIs OR “digital slides”) AND ABSTRACT:(validated OR validation OR valid OR validating OR validate). Filters: Proceedings & 2010–2022.

## Appendix E Results of internal validation of ML models

### E.1 Results of internal validation of the classification and prognostic model

Wang et al. achieved an AUC that ranged from 0.919 (95% CI 0.884–0.955) to 0.937 (95% CI 0.887–0.987) in different datasets used for internal validation when separating Nottingham Histological Grade 1 and Nottingham Histological Grade 3 invasive breast carcinomas. In addition, those predicted as high grade showed an increased risk for recurrence, with a HR of 2.94 (95% CI 1.24–6.97, *P* = .015). Pathologists’ opinions were also used as the ground truth.^41^

## References

[1] Sung H., Ferlay J., Siegel R.L. (2021). Global cancer statistics 2020: GLOBOCAN estimates of incidence and mortality worldwide for 36 cancers in 185 countries. CA Cancer J Clin..

[2] Khened M., Kori A., Rajkumar H., Krishnamurthi G., Srinivasan B. (2021). A generalized deep learning framework for whole-slide image segmentation and analysis. Sci Rep..

[3] Leong A.S.Y., Zhuang Z. (2011). The changing role of pathology in breast cancer diagnosis and treatment. Pathobiol J Immunopathol Mol Cell Biol..

[4] Nam S., Chong Y., Jung C.K. (2020). Introduction to digital pathology and computer-aided pathology. J Pathol Transl Med..

[5] Randell R., Ruddle R.A., Treanor D. (2015). Barriers and facilitators to the introduction of digital pathology for diagnostic work. Stud Health Technol Inform..

[6] Chong Y., Kim D.C., Jung C.K. (2020). Recommendations for pathologic practice using digital pathology: consensus report of the Korean Society of Pathologists. J Pathol Transl Med..

[7] Têtu B., Evans A. (2014). Canadian licensure for the use of digital pathology for routine diagnoses: one more step toward a new era of pathology practice without borders. Arch Pathol Lab Med..

[8] Azam A.S., Miligy I.M., Kimani P.K.U. (2021). Diagnostic concordance and discordance in digital pathology: a systematic review and meta-analysis. J Clin Pathol..

[9] Russell S., Norvig P. (2021).

[10] Radakovich N., Nagy M., Nazha A. (2020). Machine learning in haematological malignancies. Lancet Haematol..

[11] Norgeot B., Quer G., Beaulieu-Jones B.K. (2020). Minimum information about clinical artificial intelligence modeling: the MI-CLAIM checklist. Nat Med..

[12] Maleki F., Muthukrishnan N., Ovens K., Reinhold C., Forghani R. (2020). Machine learning algorithm validation: From essentials to advanced applications and implications for regulatory certification and deployment. Neuroimaging Clin N Am..

[13] Hastie T., Tibshirani R., Friedman J. (2017). https://hastie.su.domains/ElemStatLearn/.

[14] Homeyer A., Geißler C., Schwen L.O. (2022). Recommendations on compiling test datasets for evaluating artificial intelligence solutions in pathology. Mod Pathol..

[15] Ho S.Y., Phua K., Wong L., Bin Goh W.W. (2020). Extensions of the external validation for checking learned model interpretability and generalizability. Patterns..

[16] Nagendran M., Chen Y., Lovejoy C.A. (2020). Artificial intelligence versus clinicians: systematic review of design, reporting standards, and claims of deep learning studies. BMJ..

[17] Park S.H., Choi J., Byeon J.S. (2021). Key principles of clinical validation, device approval, and insurance coverage decisions of artificial intelligence. Korean J Radiol..

[18] Schmitt M., Maron R.C., Hekler A. (2021). Hidden variables in deep learning digital pathology and their potential to cause batch effects: prediction model study. J Med Internet Res..

[19] Kothari S., Phan J.H., Wang M.D. (2013). Eliminating tissue-fold artifacts in histopathological whole-slide images for improved image-based prediction of cancer grade. J Pathol Inform..

[20] Shmatko A., Ghaffari Laleh N., Gerstung M., Kather J.N. (2022). Artificial intelligence in histopathology: enhancing cancer research and clinical oncology. Nat Cancer..

[21] Chiriboga L., Callis G.M., Wang Y., Chlipala E. (2022). Guide for collecting and reporting metadata on protocol variables and parameters from slide-based histotechnology assays to enhance reproducibility. J Histotechnol..

[22] Adeoye J., Tan J.Y., Choi S.W., Thomson P. (2021). Prediction models applying machine learning to oral cavity cancer outcomes: a systematic review. Int J Med Inf..

[23] Akazawa M., Hashimoto K. (2021). Artificial intelligence in gynecologic cancers: current status and future challenges - a systematic review. Artif Intell Med..

[24] Bang C.S., Lee J.J., Baik G.H. (2021). Computer-aided diagnosis of esophageal cancer and neoplasms in endoscopic images: a systematic review and meta-analysis of diagnostic test accuracy. Gastrointest Endosc..

[25] Nwanosike E.M., Conway B.R., Merchant H.A., Hasan S.S. (2021). Potential applications and performance of machine learning techniques and algorithms in clinical practice: a systematic review. Int J Med Inf..

[26] Shelmerdine S.C., Arthurs O.J., Denniston A., Sebire N.J. (2021). Review of study reporting guidelines for clinical studies using artificial intelligence in healthcare. BMJ Health Care Inform..

[27] Shi Z., Zhang Z., Liu Z. (2021). Methodological quality of machine learning-based quantitative imaging analysis studies in esophageal cancer: a systematic review of clinical outcome prediction after concurrent chemoradiotherapy. Eur J Nucl Med Mol Imaging..

[28] Twilt J.J., van Leeuwen K.G., Huisman H.J., Fütterer J.J., de Rooij M. (2021). Artificial intelligence based algorithms for prostate cancer classification and detection on magnetic resonance imaging: a narrative review. Diagn Basel Switz..

[29] Page M.J., McKenzie J.E., Bossuyt P.M. (2021). The PRISMA 2020 statement: an updated guideline for reporting systematic reviews. BMJ..

[30] Ouzzani M., Hammady H., Fedorowicz Z., Elmagarmid A. (2016). Rayyan—a web and mobile app for systematic reviews. Syst Rev..

[31] Zaugg H., West R.E., Tateishi I., Randall D.L. (2011). Mendeley: creating communities of scholarly inquiry through research collaboration. TechTrends..

[32] Cano F., Madabhushi A., Cruz-Roa A. (2018). 14th International Symposium on Medical Information Processing and Analysis.

[33] Cruz-Roa A., Gilmore H., Basavanhally A. (2017). Accurate and reproducible invasive breast cancer detection in whole-slide images: a deep learning approach for quantifying tumor extent. Sci Rep..

[34] Cruz-Roa A., Gilmore H., Basavanhally A. (2018). High-throughput adaptive sampling for whole-slide histopathology image analysis (HASHI) via convolutional neural networks: Application to invasive breast cancer detection. PloS One..

[35] Colon-Cartagena L., Gayhart M., Robila V. (2020). Modern Pathology.

[36] Mi W., Li J., Guo Y. (2021). Deep learning-based multi-class classification of breast digital pathology images. Cancer Manag Res..

[37] Radiya-Dixit E., Zhu D., Beck A.H. (2017). Automated Classification of benign and malignant proliferative breast lesions. *Sci Rep*..

[38] Yang Z., Ran L., Zhang S., Xia Y., Zhang Y. (2019). EMS-Net: Ensemble of multiscale convolutional neural networks for classification of breast cancer histology images. *Neurocomputing*..

[39] Bai Y., Cole K., Martinez-Morilla S. (2021). An open-source, automated tumor-infiltrating lymphocyte algorithm for prognosis in triple-negative breast cancer. Clin Cancer Res..

[40] Bychkov D., Joensuu H., Nordling S. (2022). Outcome and biomarker supervised deep learning for survival prediction in two multicenter breast cancer series. J Pathol Inform..

[41] Wang Y., Acs B., Robertson S. (2022). Improved breast cancer histological grading using deep learning. *Ann Oncol*..

[42] Moons K.G.M., Wolff R.F., Riley R.D. (2019). PROBAST: a tool to assess risk of bias and applicability of prediction model studies: explanation and elaboration. Ann Intern Med..

[43] Kim D.W., Jang H.Y., Kim K.W., Shin Y., Park S.H. (2019). Design characteristics of studies reporting the performance of artificial intelligence algorithms for diagnostic analysis of medical images: results from recently published papers. Korean J Radiol..

[44] Nguyen A.V., Blears E.E., Ross E., Lall R.R., Ortega-Barnett J. (2018). Machine learning applications for the differentiation of primary central nervous system lymphoma from glioblastoma on imaging: a systematic review and meta-analysis. Neurosurg Focus..

[45] Yao A.D., Cheng D.L., Pan I., Kitamura F. (2020). Deep learning in neuroradiology: a systematic review of current algorithms and approaches for the new wave of imaging technology. Radiol Artif Intell..

[46] Corti C., Cobanaj M., Marian F. (2022). Artificial intelligence for prediction of treatment outcomes in breast cancer: systematic review of design, reporting standards, and bias. Cancer Treat Rev..

[47] Mazo C., Aura C., Rahman A., Gallagher W.M., Mooney C. (2022). Application of artificial intelligence techniques to predict risk of recurrence of breast cancer: a systematic review. J Pers Med..

[48] Yu A.C., Mohajer B., Eng J. (2022). External validation of deep learning algorithms for radiologic diagnosis: A systematic review. Radiol Artif Intell..

[49] Collins G.S., Reitsma J.B., Altman D.G., Moons K.G.M. (2015). Transparent reporting of a multivariable prediction model for individual prognosis or diagnosis (TRIPOD): the TRIPOD statement. Ann Intern Med..

[50] Luo W., Phung D., Tran T. (2016). Guidelines for developing and reporting machine learning predictive models in biomedical research: a multidisciplinary view. J Med Internet Res..

[51] Park S.H., Han K. (2018). Methodologic guide for evaluating clinical performance and effect of artificial intelligence technology for medical diagnosis and prediction. Radiology..

[52] Yu K.H., Wang F., Berry G.J. (2020). Classifying non-small cell lung cancer types and transcriptomic subtypes using convolutional neural networks. J Am Med Inform Assoc..

[53] Gildenblat J, Klaiman E. Self-Supervised Similarity Learning for Digital Pathology. Published online January 13, 2020. 10.48550/arXiv.1905.08139

[54] Abu Haeyeh Y., Ghazal M., El-Baz A., Talaat I.M. (2022). Development and evaluation of a novel deep-learning-based framework for the classification of renal histopathology images. Bioengineering..

[55] Janowczyk A., Baumhoer D., Dirnhofer S. (2022). Towards a national strategy for digital pathology in Switzerland. Virchows Arch..

[56] Komura D., Ishikawa S. (2018). Machine learning methods for histopathological image analysis. Comput Struct Biotechnol J..

[57] Schüffler P.J., Geneslaw L., Yarlagadda D.V.K. (2021). Integrated digital pathology at scale: a solution for clinical diagnostics and cancer research at a large academic medical center. J Am Med Inform Assoc..

[58] Zehra T., Shabbir A. (2021). Adoption of digital pathology in developing countries: from benefits to challenges. J Coll Physicians Surg Pak..

[59] Pantanowitz L., Sharma A., Carter A.B., Kurc T., Sussman A., Saltz J. (2018). Twenty years of digital pathology: an overview of the road travelled, what is on the horizon, and the emergence of vendor-neutral archives. J Pathol Inform..

[60] Cooper M., Ji Z., Krishnan R.G. (2023). Machine learning in computational histopathology: challenges and opportunities. Genes Chromosomes Cancer..

[61] Savage N. (2020). The race to the top among the world’s leaders in artificial intelligence. *Nature*..

[62] Fell C., Mohammadi M., Morrison D., Arandjelovic O., Caie P., Harris-Birtill D. (2022). Reproducibility of deep learning in digital pathology whole slide image analysis. PLoS Digit Health..

[63] Gao Y., Li S., Jin Y. (2021). Assessment of performance of the machine learning-based breast cancer risk prediction model: a systematic review and meta-analysis (preprint). JMIR Public Health Surveil.

[64] Dhiman P., Ma J., Navarro C.A. (2021). Reporting of prognostic clinical prediction models based on machine learning methods in oncology needs to be improved. J Clin Epidemiol..

[65] Sounderajah V., Ashrafian H., Aggarwal R. (2020). Developing specific reporting guidelines for diagnostic accuracy studies assessing AI interventions: the STARD-AI Steering Group. Nat Med..

[66] Vasey B., Nagendran M., Campbell B. (2022). Reporting guideline for the early-stage clinical evaluation of decision support systems driven by artificial intelligence: DECIDE-AI. Nat Med..

[67] Blanco D., Altman D., Moher D., Boutron I., Kirkham J.J., Cobo E. (2019). Scoping review on interventions to improve adherence to reporting guidelines in health research. BMJ Open..

[68] Zormpas-Petridis K., Noguera R., Ivankovic D.K., Roxanis I., Jamin Y., Yuan Y. (2020). SuperHistopath: a deep learning pipeline for mapping tumor heterogeneity on low-resolution whole-slide digital histopathology images. Front Oncol..

[69] Tellez D., Litjens G., Bándi P. (2019). Quantifying the effects of data augmentation and stain color normalization in convolutional neural networks for computational pathology. Med Image Anal..

[70] Boschman J., Farahani H., Darbandsari A. (2022). The utility of color normalization for AI-based diagnosis of hematoxylin and eosin-stained pathology images. J Pathol..

[71] Khan S., Rahmani H., Shah S.A.A., Bennamoun M., Khan S., Rahmani H., Shah S.A.A., Bennamoun M. (2018). A Guide to Convolutional Neural Networks for Computer Vision.

[72] Iglesias L.L., Bellón P.S., del Barrio A.P. (2021). A primer on deep learning and convolutional neural networks for clinicians. Insights Imaging..

[73] Yamashita R., Nishio M., Do R.K.G., Togashi K. (2018). Convolutional neural networks: an overview and application in radiology. Insights Imaging..

[74] Dosovitskiy A, Beyer L, Kolesnikov A, et al. An Image is Worth 16x16 Words: Transformers for Image Recognition at Scale. Published online June 3, 2021. 10.48550/arXiv.2010.11929

[75] Dhiman P., Ma J., Andaur Navarro C.L. (2022). Risk of bias of prognostic models developed using machine learning: a systematic review in oncology. Diagn Progn Res..

[76] Van Calster B., Steyerberg E.W., Wynants L., van Smeden M. (2023). There is no such thing as a validated prediction model. BMC Med..

[77] Tang H., Sun N., Shen S. (2021). Improving generalization of deep learning models for diagnostic pathology by increasing variability in training data: experiments on osteosarcoma subtypes. J Pathol Inform..

[78] Vali-Betts E., Krause K.J., Dubrovsky A. (2021). Effects of image quantity and image source variation on machine learning histology differential diagnosis models. J Pathol Inform..

[79] The College of American Pathologists. How to Validate AI Algorithms in Anatomic Pathology. College of American Pathologists. Published 2019. Accessed May 7, 2023. https://www.cap.org/member-resources/clinical-informatics-resources/how-to-validate-ai-algorithms-in-anatomic-pathology

[80] U.S. Food and Drug Administration, Center for Devices and Radiological Health (CDRH) DEN200080. De Novo Request for Classification or the Paige Prostate. https://www.accessdata.fda.gov/cdrh_docs/pdf20/DEN200080.pdf.

[81] Rojas J.C., Fahrenbach J., Makhni S. (2022). Framework for integrating equity into machine learning models: a case study. CHEST..

[82] Shankar S, Herman B, Parameswaran AG. Rethinking Streaming Machine Learning Evaluation. Published online May 23, 2022. 10.48550/arXiv.2205.11473

[83] Symeonidis G., Nerantzis E., Kazakis A., Papakostas G.A. (2022). 2022 IEEE 12th Annual Computing and Communication Workshop and Conference (CCWC).

[84] Liu G., Xu T., Ma X., Wang C. (2022). Your model trains on my data? Protecting intellectual property of training data via membership fingerprint authentication. IEEE Trans Inf Forensics Secur..

[85] WHO Blue Books Web Site Launched – IARC. IARC News. Accessed February 13, 2023. https://www.iarc.who.int/news-events/who-blue-books-web-site-launched/

[86] Cserni G. (2020). Histological type and typing of breast carcinomas and the WHO classification changes over time. *Pathologica*..

[87] Sandbank J., Bataillon G., Nudelman A. (2022). Validation and real-world clinical application of an artificial intelligence algorithm for breast cancer detection in biopsies. Npj Breast Cancer..

[88] Chan R.C., To CKC, Cheng K.C.T., Yoshikazu T., Yan L.L.A., Tse G.M. (2023). Artificial intelligence in breast cancer histopathology. Histopathology..

[89] MEDLINE® 2023 Database Guide. Ovid Database Guide. https://ospguides.ovid.com/OSPguides/medline.htm.

[90] Hoeppner M.A. (2013). NCBI bookshelf: books and documents in life sciences and health care. Nucleic Acids Res..

[91] Embase: Excerpta Medica Database Guide. Ovid Database Guide. https://ospguides.ovid.com/OSPguides/embase.htm.

[92] CINAHL Database | EBSCO. https://www.ebsco.com/products/research-databases/cinahl-database.

[93] About IEEE Xplore. https://ieeexplore.ieee.org/Xplorehelp/overview-of-ieee-xplore/about-ieee-xplore.

[94] MICCAI Society Publications. http://www.miccai.org/publications/.

[95] MICCAI Society Events. http://www.miccai.org/events/.

[96] Proceedings on SPIE Digital Library. https://www.spiedigitallibrary.org/conference-proceedings-of-spie?webSyncID=9441a392-2138-922d-be78-38e4ea5e2c5a&sessionGUID=a00d5892-1dbb-6bd0-83b3-094c0cd11686&_ga=2.19419128.1587339309.1694992598-2003971750.1694992598&cm_mc_uid=01701106380816949925985&cm_mc_sid_50300000=65096671694996533306&SSO=1.

